# Rebound After Guided Growth for Idiopathic Genu Valgum: Long-Term Radiographic Follow-Up to Skeletal Maturity and Associated Factors

**DOI:** 10.3390/jcm15166367

**Published:** 2026-08-18

**Authors:** Philipp Scheider, Ghanem El-Isa, Andreas Kranzl, Sebastian Farr

**Affiliations:** 1 Department of Pediatric Orthopaedics and Foot and Ankle Surgery, Orthopaedic Hospital Speising, 1130 Vienna, Austria; ghanem.el_isa@hotmail.com; 2 Medical Campus, Medical University of Vienna, 1090 Vienna, Austria; 3 Laboratory for Gait and Movement Analysis, Orthopaedic Hospital Speising, 1130 Vienna, Austria; andreas.kranzl@oss.at

**Keywords:** genu valgum, guided growth, tension-band plate, hemiepiphysiodesis, rebound, mechanical axis deviation, skeletal maturity

## Abstract

**Background/Objectives:** Temporary hemiepiphysiodesis corrects idiopathic genu valgum during growth, but alignment may drift after implant removal. Radiographic alignment to skeletal maturity was evaluated, and factors associated with post-removal rebound were explored. **Methods:** This single-center longitudinal observational cohort study included 17 patients (31 limbs) treated with tension-band plates. Standing long-leg radiographs were assessed before implantation (t0), at plate removal (t1), and at final follow-up after skeletal maturity (t2). Rebound was defined as a lateral mechanical-axis deviation (MAD) shift of at least 5 mm from t1 to t2. Paired comparisons and exploratory linear regression models were used to assess longitudinal changes and potential predictors of rebound. **Results:** Mean age was 13.53 ± 1.27 years at t0, 14.67 ± 1.45 years at t1, and 19.64 ± 1.08 years at t2. MAD changed from −15.06 ± 7.32 mm at t0 to 3.65 ± 7.29 mm at t1 and −1.03 ± 7.48 mm at t2. The mean lateral shift after plate removal was 4.68 ± 6.55 mm (*p* < 0.001). A total of 15 of 31 limbs (48.4%) met the rebound definition. At t2, 29 limbs (93.5%) remained within Stevens zones ±1. Greater residual growth (univariate *b* = 0.621, *p* = 0.003) and isolated distal femoral plating (univariate *b* = 5.534 mm, *p* = 0.017) were associated with greater lateral drift and remained significant in the multivariable model. **Conclusions:** Post-removal rebound was frequent but generally modest, with the observed alignment drift predominantly reflecting distal femoral change. Nevertheless, most limbs remained within the central, clinically acceptable Stevens zones at skeletal maturity. These findings support continued radiographic surveillance, particularly following isolated distal femoral correction and in patients with substantial residual growth.

## 1. Introduction

Persistent idiopathic genu valgum beyond the physiologic age range may cause symptoms, alter lower-limb mechanics, and raise concern about long-term joint loading [1]. Temporary hemiepiphysiodesis with tension-band plates is widely used because it permits gradual correction while avoiding an acute osteotomy in skeletally immature patients [2,3]. Radiographic alignment remains the principal structural outcome, although static measures do not fully reflect dynamic gait mechanics [4,5]. The procedure is technically straightforward, but its success depends on implant timing, correction rate, remaining growth, and alignment behavior after implant removal [3].

Post-removal rebound remains one of the least predictable parts of guided growth. Reported definitions and incidences vary substantially, and proposed risk factors include young age, rapid correction, greater remaining growth, and implant location [6,7,8,9,10,11,12,13,14,15]. A systematic review found that follow-up practices were heterogeneous and emphasized surveillance until skeletal maturity [6]. Recent work has also suggested that static and dynamic measures may provide complementary information when recurrent deformity is considered [16].

Published short-term data demonstrate effective frontal-plane correction at the time of implant removal and concurrent changes in knee mechanics [5]. However, most guided-growth studies emphasize the correction achieved at removal, whereas follow-up thereafter is shorter, heterogeneous, or not consistently continued to skeletal maturity [6,7,8,9,10,11,12,13,14,15]. Consequently, it remains unclear whether correction is maintained through the remaining growth period, which anatomic segment contributes most to a later alignment shift, and which clinical or treatment-related factors are associated with rebound. A three-time-point evaluation from implantation through removal to skeletal maturity is therefore required to assess long-term radiographic effectiveness.

The primary objective was to describe the radiographic course from implantation through plate removal to final follow-up after growth completion. Secondary objectives were to quantify rebound after plate removal and to explore clinical and treatment-related factors associated with the magnitude of lateral mechanical-axis deviation (MAD) drift.

## 2. Materials and Methods

### 2.1. Study Design and Patient Selection

The single-center longitudinal observational cohort study was conducted at the Orthopaedic Hospital Speising, Vienna, Austria. The study was approved by the responsible ethics committee (EK24/2020), and written informed consent was obtained before the final follow-up assessment. Demographic data, treatment-related parameters, and radiographic measurements were recorded. Reporting was organized according to the STROBE principles.

Potentially eligible participants were retrospectively identified from the institutional database and invited to undergo a standardized final assessment after skeletal maturity. Preoperative (t0) and plate-removal data (t1) were obtained from existing records, whereas the final clinical and radiographic assessments (t2) were prospectively performed.

Eligibility required pre-correction and post-correction standing long-leg radiographs and age older than 18 years at final follow-up. Neuromuscular disease, skeletal dysplasia, syndromic deformity, failure to achieve correction, relevant limb-length discrepancy, recurrent deformity requiring repeat plate implantation, and major subsequent surgery of the same limb were exclusion criteria.

Patients who underwent repeat guided growth were excluded because reintervention interrupted the prespecified untreated t1-to-t2 observation interval and precluded assessment of the post-removal alignment course under the original treatment alone.

**Figure 1:**
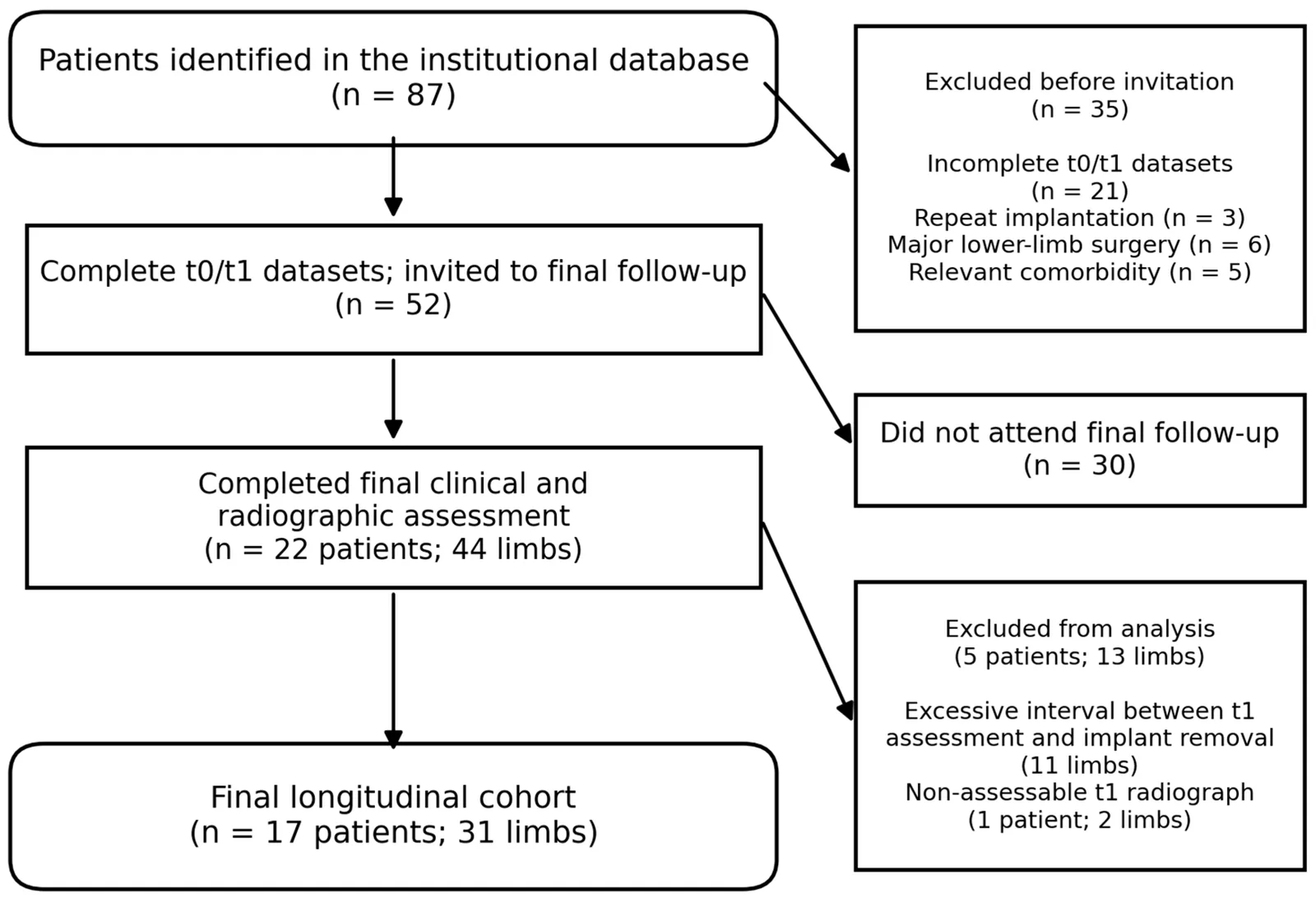
Flowchart of patient recruitment and study cohort selection.

Eighty-seven potentially eligible patients were identified. Five were excluded for comorbid conditions, three for repeat plate implantation because of recurrent genu valgum, and six for major additional lower-limb surgery. Of 52 invited patients with a complete earlier dataset, 22 consented to final evaluation. Eleven limbs were subsequently excluded because the interval between the postoperative investigations and actual implant removal was too long; one additional patient was excluded because the postoperative radiograph was not assessable. The final analysis comprised 17 patients and 31 treated limbs. The patient recruitment process is illustrated in Figure 1.

### 2.2. Treatment and Follow-Up

Tension-band plates were placed across the distal medial femoral physis, proximal medial tibial physis, or both, according to the deformity origin. Assessment time points were defined as preoperative/implantation (t0), implant removal (t1), and final follow-up after skeletal maturity (t2).

Residual longitudinal growth was defined as the increase in standing height from t1 to t2. Chronological age was analyzed separately and was not included in this calculation. Because skeletal age was not assessed, observed height gain served as a proxy for growth during the post-removal follow-up period.

### 2.3. Radiographic Assessment

Anteroposterior weight-bearing full-length radiographs were obtained with both patellae facing forward, the femoral condyles parallel to the frontal plane, and a 25 mm calibration sphere positioned close to the femur. All digital measurements were performed with the TraumaCad software, version 2.5 (Brainlab Inc., Munich, Germany), by the same study investigator. The hip, knee, and ankle centers were identified to construct the mechanical axes. The recorded parameters were MAD, mechanical lateral distal femoral angle (mLDFA), mechanical medial proximal tibial angle (mMPTA), mechanical tibiofemoral angle (mTFA), mechanical lateral distal tibial angle (mLDTA), mechanical lateral proximal femoral angle (mLPFA), and joint-line convergence angle (JLCA) [17,18,19,20].

MAD was defined as the distance at knee level between the knee center and the mechanical limb axis connecting the center of the femoral head with the center of the ankle. Positive values indicated medial deviation and negative values lateral deviation. The Paley reference for skeletally mature limbs was 8 ± 7 mm medial to the knee center [17]. The mLDFA and mMPTA were used to identify femoral and tibial contributions to malalignment, respectively, while JLCA characterized joint-line convergence. Paley reference ranges used for interpretation were 85–90° for mLDFA, 85–90° for mMPTA, 86–92° for mLDTA, 85–95° for mLPFA, and 0–2° of medial convergence for JLCA [17,18]. Positive mTFA values indicated valgus and negative values varus.

**Figure 2:**
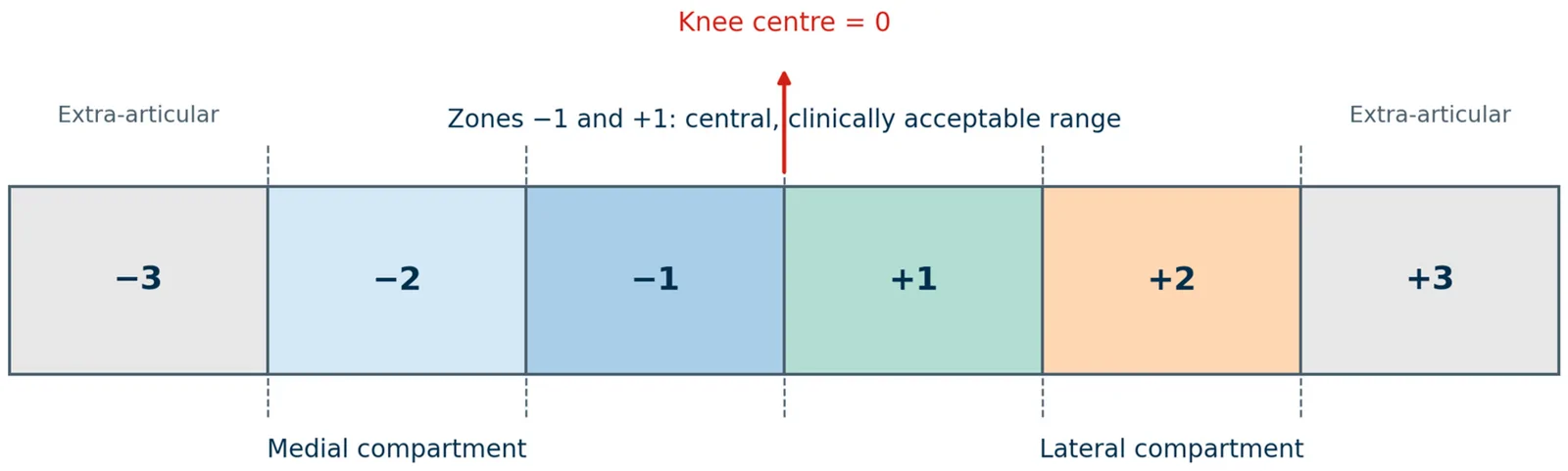
Schematic AP knee representation of the Stevens mechanical-axis zones. The knee center is designated as zero; the inner medial and lateral halves are zones −1 and +1; the outer halves are zones −2 and +2; and extra-articular courses are zones −3 and +3.

Mechanical-axis location was additionally categorized using the Stevens zone system [21]. The knee center was designated as zero. The inner halves of the medial and lateral compartments were classified as zones −1 and +1, the outer halves as zones −2 and +2, and an extra-articular mechanical-axis course as zones −3 and +3 (Figure 2). Zones −1 and +1 were regarded as the central, clinically acceptable region. This width-normalized classification was used as a descriptive complement to continuous MAD and not as the primary rebound definition.

Rebound was defined as a lateral MAD shift of at least 5 mm between t1 and t2. The continuous t1-to-t2 MAD change was used as the outcome in exploratory predictor analyses. Correction rates were calculated as the absolute t0-to-t1 change divided by the interval between assessments in months. MAD and mTFA rates were calculated for the complete cohort; mLDFA rates were calculated for limbs treated at the distal femur and mMPTA rates for limbs treated at the proximal tibia.

### 2.4. Statistical Analysis

Descriptive and inferential statistical analyses were performed using IBM SPSS Statistics, version 20.0 (IBM Deutschland GmbH, Ehningen, Germany). A *p*-value ≤ 0.05 was considered statistically significant.

Descriptive statistics for continuous variables were presented as mean ± standard deviation. For categorical variables, absolute frequencies and percentages were reported. Radiographic t1-to-t2 changes were tested with paired *t*-tests after Shapiro–Wilk assessment of normality. Potential predictors of rebound magnitude were first assessed in univariable linear regression. Variables with *p* ≤ 0.05 were considered for a backward multivariable model.

Because the number of observations and candidate predictors was limited, the regression analyses were regarded as exploratory. Limbs served as observational units, and within-patient correlation between bilaterally treated limbs was not modeled.

## 3. Results

### 3.1. Cohort Characteristics

The cohort included 11 male and 6 female patients; 14 underwent bilateral and 3 unilateral correction. Seventeen limbs received isolated distal femoral plating, nine isolated proximal tibial plating, and five combined femoral and tibial plating. Mean implant duration was 13.66 ± 8.58 months; mean follow-up from removal to t2 was 59.66 ± 12.13 months. Residual longitudinal growth after removal averaged 7.6 ± 5.5 cm. Demographic and treatment-related data are summarized in Table 1.

### 3.2. Radiographic Course

Mean MAD corrected by 18.71 ± 8.65 mm between implantation and removal. Mean correction rates during the implant period were 2.18 ± 1.89 mm/month for MAD and 0.51 ± 0.37°/month for mTFA. Segment-specific angular correction rates were 0.31 ± 0.36°/month for mLDFA in femorally treated limbs and 0.12 ± 0.26°/month for mMPTA in tibially treated limbs. From t1 to t2, the mechanical axis shifted laterally by 4.68 ± 6.55 mm (paired t(30) = 3.97, *p* < 0.001), yielding a net t0-to-t2 correction of 14.03 ± 10.45 mm. The mLDFA decreased significantly after plate removal, whereas the mMPTA did not change significantly, indicating that the average rebound was primarily femoral. The mTFA also shifted toward valgus between t1 and t2 (Table 2).

### 3.3. Rebound and Final Alignment

Fifteen limbs (48.4%) demonstrated a lateral MAD shift of at least 5 mm and, therefore, fulfilled the predefined criterion for rebound. One limb shifted medially by 10 mm, whereas the remaining fifteen limbs showed no relevant MAD change. At final follow-up, 29 of 31 limbs (93.5%) remained within the central Stevens zones (−1 and +1), while 10 limbs (32.3%) fell within the Paley reference range for MAD. The distribution of rebound according to implant location is summarized in Table 3.

### 3.4. Factors Associated with Lateral MAD Drift

Compared with isolated distal femoral plating, isolated proximal tibial plating was associated with 5.621 mm less lateral drift (95% CI, 0.441–10.800; *p* = 0.034). In univariable models, isolated distal femoral treatment and residual growth were associated with greater rebound. A shorter correction duration was also associated with a greater lateral drift (*b* = −0.285, *p* = 0.039) but was not retained in the final model. Chronological age at t0 or t1, sex, initial valgus magnitude, and MAD correction rate were not significant predictors. Isolated femoral treatment and residual growth remained significant in the multivariable model (Table 4).

Given the limited sample, the multivariable coefficients are presented as exploratory associations rather than definitive risk estimates.

## 4. Discussion

The principal finding was that lower-limb alignment drifted modestly toward valgus after plate removal, and almost half of the limbs met the prespecified ≥5 mm rebound definition. The change was statistically significant and predominantly femoral: mLDFA decreased between t1 and t2, whereas mMPTA remained stable. Nevertheless, mean mLDFA and mTFA remained within commonly used physiological ranges, and 29 of 31 limbs remained within Stevens zones ±1 at skeletal maturity.

**Figure 3:**
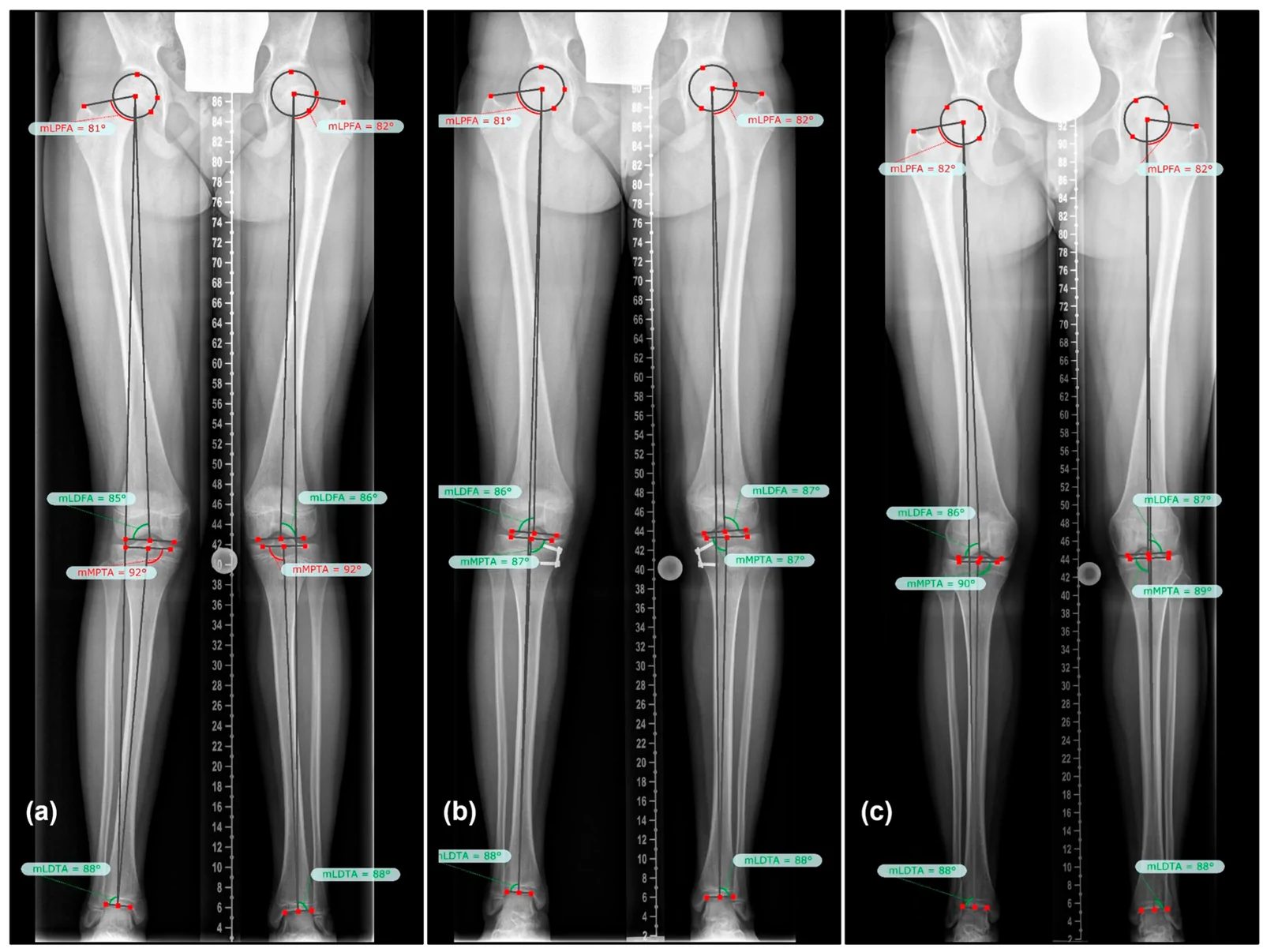
Radiographic course of a patient treated with bilateral proximal medial tibial tension-band plating for guided growth. Standing anteroposterior long-leg radiographs with mechanical-axis lines and joint-orientation angle measurements are shown: (**a**) preoperatively (t0), demonstrating bilateral valgus malalignment; (**b**) at the time of implant removal (t1), showing correction of the mechanical-axis deviation and joint-orientation angles; (**c**) at final follow-up after skeletal maturity (t2), demonstrating recurrent lateral mechanical-axis deviation consistent with rebound.

The correction achieved by plate removal was consistent with published series of tension-band plating. Burghardt and Herzenberg reported successful correction in 50 of 54 treated bone segments (90%), Dai et al. reported correction of 94% of coronal deformities, and Danino et al. achieved physiological segmental alignment in 93% of femoral and 92% of tibial deformities [8,22,23]. In the present cohort, 22 of 31 limbs (71%) met the Paley MAD reference at removal. However, 30 of 31 limbs (97%) were within Stevens zones ±1 at removal and 29 of 31 (94%) remained within these zones at skeletal maturity (Figure 3). The latter proportions are comparable with the published correction rates, although the outcome definitions are not identical.

The difference between Paley- and Stevens-based success rates reflects the underlying measurement concepts. Paley defined an adult MAD reference of 8 ± 7 mm medial to the knee center, which excludes an axis passing exactly through the center or slightly lateral to it [17]. The Stevens classification instead normalizes axis location to knee width and regards the inner medial and lateral halves (zones −1 and +1) as the central reference region [21]. This approach is useful for clinical surveillance and accommodates differences in knee size and radiographic magnification. Continuous MAD remains preferable for quantifying rebound because an ordinal zone may change after a minimal shift near a boundary [17,18,20,21].

Reported rebound frequencies vary widely because studies differ in diagnosis, implant type, overcorrection strategy, follow-up duration, and outcome definition. Stevens reported recurrence requiring repeat guided growth in 8 of 32 idiopathic valgus limbs after a mean follow-up of 14 months, but did not specify a numerical rebound threshold [2]. Using a MAD shift of more than 3 mm toward the original deformity, Farr et al. identified rebound in 25 of 58 idiopathic valgus limbs after a mean follow-up of 39.1 months [12]. Leveille et al. reported rebound in 35 of 67 varus and valgus limbs using an mTFA change greater than 5° [11]. With a threshold of at least 5° for mLDFA or mMPTA, Ramazanov et al. observed rebound in 56% of valgus and 24% of varus segments, whereas Choi et al. reported corresponding frequencies of 45% and 39% [9,10]. In contrast, Dai et al. observed rebound in only 3 of 101 limbs in children younger than 10 years old; however, prophylactic overcorrection was applied, rebound was defined as a deviation of more than 3° from the stated angular norm, and mean follow-up was only 12.7 months [8].

Given this methodological heterogeneity, the observed rebound frequency of 48.4% (15 of 31 limbs) should be interpreted as the proportion of analyzed limbs showing a lateral MAD drift of at least 5 mm rather than as a treatment-failure rate. This frequency was comparable to rates in studies applying similar MAD or angular thresholds [9,10,12], whereas the markedly lower rate reported by Dai et al. was observed following prophylactic overcorrection and a substantially shorter follow-up period [8]. The 5 mm cutoff was an operational radiographic threshold for longitudinal MAD change: although 15 limbs crossed it, 29 of 31 remained within Stevens zones ±1 at skeletal maturity, indicating that most threshold-defined shifts did not move alignment outside the central Stevens region.

The 48.4% estimate applies only to the cohort completing the t1-to-t2 interval without repeat guided growth. The three excluded patients requiring repeat plate implantation had clinically important recurrence; their exclusion may therefore have underestimated severe recurrence and shifted the reported frequency and predictor estimates toward more favorable outcomes. A direct comparison of radiographic rebound with symptoms or function was not possible because t0 and t1 were assessed retrospectively and standardized clinical outcomes were not available at all three time points.

Associations with age, body mass index, and initial deformity have been inconsistent. Park et al. suggested a protective association with higher body mass index, whereas Farr et al. reported a 12% increase in rebound risk for each additional body mass index unit; Choi et al. found no significant association [7,10,12]. An inverse association with body mass index at t2 was observed in the univariable analysis (*b* = −0.534, *p* = 0.048), but this postoperative measure was not entered into the multivariable model and should not be interpreted causally. Park et al. and Leveille et al. associated greater initial deformity with recurrence, whereas initial valgus magnitude was not a significant predictor in the present cohort [7,11]. Younger age has repeatedly been proposed as a risk factor [2,7,9,11,12,24], but neither implantation nor removal age was significant in this case. Chronological age may be an imprecise surrogate for physeal activity when skeletal age is unavailable.

Correction rate may provide a more direct measure of physeal activity. Park et al. reported that 11 of 14 rebound cases (77%) had corrected at ≥8.5° per year, and Choi et al. estimated a 20% increase in rebound risk for each additional degree per year of mLDFA or mMPTA correction [7,10]. Ballal et al., Dai et al., Ramazanov et al., and Burghardt and Herzenberg also described faster correction in younger patients or at the distal femur [8,9,22,25]. In the present cohort, mean mLDFA correction was 0.31° per month in femorally treated limbs compared with 0.12° per month for mMPTA in tibially treated limbs. These segment-specific rates were descriptive and were not formally compared. Only MAD correction rate entered the predictor analysis and was not significant, whereas shorter correction duration was associated with greater lateral drift in the univariable model.

**Figure 4:**
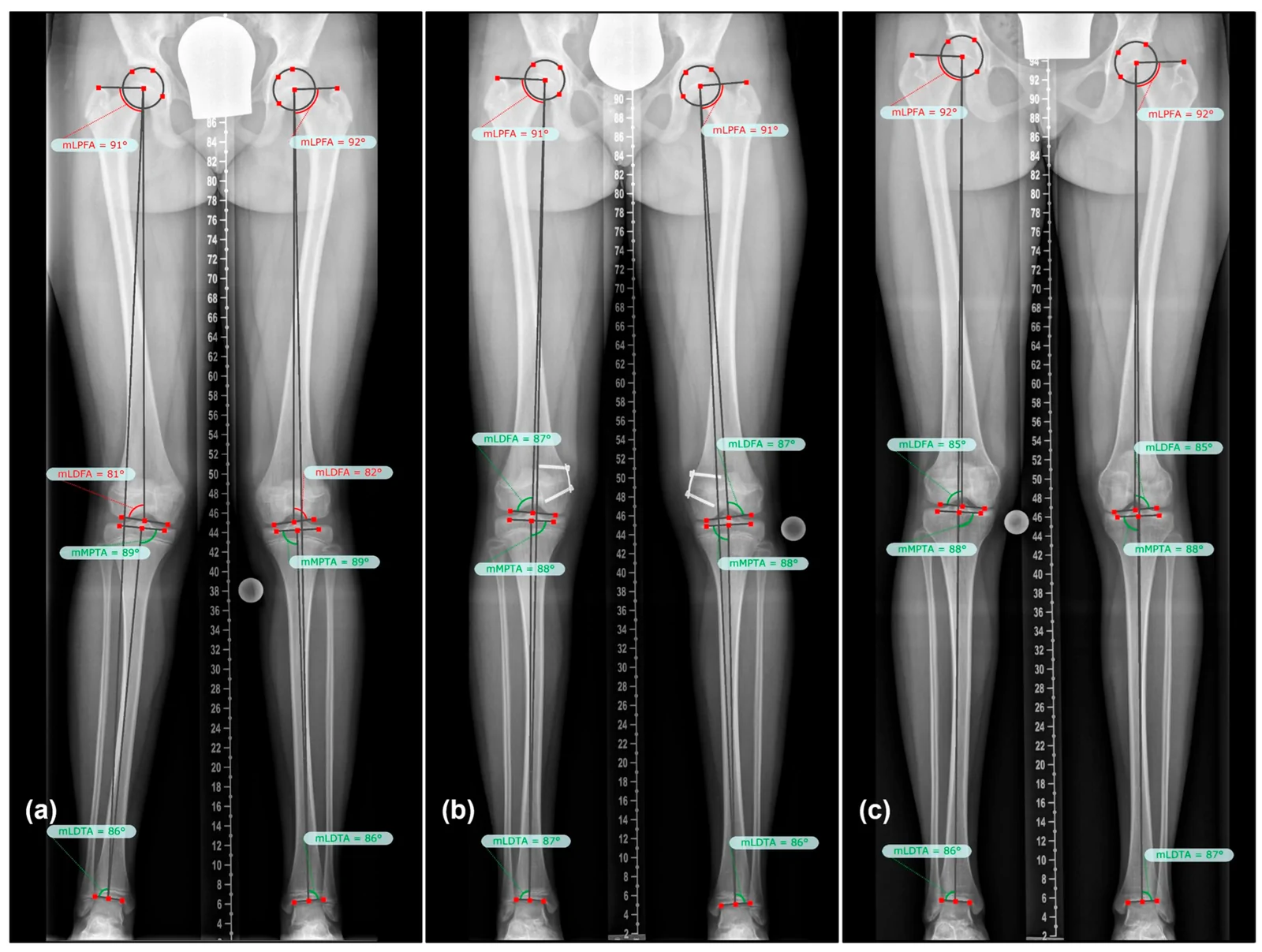
Radiographic course of a patient treated with bilateral distal medial femoral tension-band plating for guided growth. Standing anteroposterior long-leg radiographs with mechanical-axis lines and joint-orientation angle measurements are shown: (**a**) valgus malalignment before treatment (t0); (**b**) correction of mechanical-axis deviation and distal femoral joint orientation at implant removal (t1); (**c**) recurrent lateral mechanical-axis deviation at skeletal maturity (t2), consistent with post-removal rebound.

Residual growth and treatment location showed the clearest associations with later drift. Each additional centimeter of growth after implant removal was associated with 0.621 mm greater lateral MAD shift. Eastwood and Sanghrajka and Burghardt and Herzenberg similarly linked longer remaining growth with rebound, while Farr et al. reported a 54% increase in risk for each additional year of remaining growth [12,22,26]. Experimental work by Ding et al. demonstrated increased physeal cellular activity after release of unilateral growth restraint, providing a plausible mechanism for post-removal catch-up growth [27]. Isolated distal femoral plating was also associated with greater drift, consistent with the faster femoral correction rate and the larger contribution of the distal femoral physis (Figure 4). Treatment location was selected according to deformity origin, however, and may partly reflect underlying anatomy rather than an independent causal effect.

In this study, residual growth described observed post-removal standing-height gain rather than bone-age-based remaining growth potential at t1. Consequently, the coefficient of 0.621 mm per centimeter relates MAD drift to subsequent height gain and cannot be translated directly into a skeletal-age-specific risk estimate. Height measurement variability and individual differences in maturation and body proportions may have influenced its magnitude and precision; the association should therefore be regarded as exploratory, while still supporting a relationship between post-removal growth and subsequent lateral MAD drift.

These findings illustrate the timing dilemma of guided growth. Treatment must begin early enough to achieve correction, yet implant removal while substantial growth remains may increase the risk of rebound. Routine overcorrection cannot be recommended based on the present data because the optimal degree of overcorrection remains unknown, and persistent varus deformity may occur in patients who do not subsequently experience rebound [3,7,10,11]. Instead, close radiographic follow-up should be considered after implant removal, particularly following isolated distal femoral correction, rapid angular correction, or implant removal in patients with substantial remaining growth. Serial standing radiographs may facilitate the early detection of clinically relevant recurrent deformity while repeat guided growth remains feasible.

Several limitations affect the conclusions. The small, selected cohort, and substantial loss to follow-up limit generalizability, while exclusion of patients requiring repeat guided growth may have underestimated severe recurrence. Residual growth was derived from observed standing-height gain rather than skeletal-age assessment; consequently, it represents height gain during follow-up rather than a direct estimate of remaining physeal growth potential at t1 and cannot be translated into a skeletal-age-specific risk estimate. The exploratory regression model included 31 limbs, used backward selection, and did not account for within-patient correlation in bilaterally treated participants. These constraints should be considered when interpreting the magnitude and precision of the estimated associations. Standardized clinical outcomes were not available at all three time points because t0 and t1 data were retrospective, precluding direct clinical–radiographic correlation. Finally, conventional anteroposterior radiographs are sensitive to limb positioning, although strictly standardized images were used to reduce this variability.

## 5. Conclusions

Tension-band plating achieved substantial correction of idiopathic genu valgum, although a modest, predominantly femoral alignment drift was observed after implant removal. Exploratory analyses suggested that greater residual growth after plate removal and isolated distal femoral treatment were associated with more pronounced rebound. Nevertheless, most limbs remained within the central, clinically acceptable Stevens zones at skeletal maturity. These findings support continued radiographic surveillance until growth completion, particularly following isolated distal femoral correction and in patients with substantial remaining growth.

## Figures and Tables

**Table 1:** Demographic and treatment characteristics.

Variable	t0	t1	t2/Final
Age, years	13.53 ± 1.27	14.67 ± 1.45	19.64 ± 1.08
Height, cm	169.4 ± 10.1	174.8 ± 10.2	182.4 ± 10.3
Weight, kg	63.6 ± 14.8	68.1 ± 15.6	84.3 ± 18.9
Body mass index, kg/m^2^	22.1 ± 4.1	22.2 ± 3.9	25.3 ± 4.8
Interval	—	13.66 ± 8.58 months from t0	59.66 ± 12.13 months from t1

Values are presented as mean ± SD.

**Table 2:** Radiographic alignment at the three assessment time points.

Parameter	t0	t1	t2	*p* (t1 vs. t2)
MAD, mm	−15.06 ± 7.32	3.65 ± 7.29	−1.03 ± 7.48	<0.001 *
mLDFA, °	84.61 ± 2.14	88.19 ± 3.12	87.06 ± 2.73	0.004 *
mMPTA, °	90.94 ± 2.32	89.74 ± 2.02	90.23 ± 2.20	0.066
mTFA, °	5.03 ± 1.91	−0.10 ± 2.47	1.68 ± 2.41	<0.001 *
mLDTA, °	86.19 ± 3.05	86.84 ± 3.75	87.48 ± 2.62	0.176
mLPFA, °	85.29 ± 3.63	85.97 ± 3.62	87.55 ± 3.30	<0.001 *
JLCA, °	1.84 ± 1.73	2.03 ± 1.72	1.81 ± 1.62	0.354

Values are presented as mean ± SD. Negative MAD values indicate lateral deviation; positive mTFA values indicate valgus. Statistically significant values are in bold. * *p* ≤ 0.05.

**Table 3:** Rebound according to implant location.

Implant Location	Limbs, *n*	Mean t1–t2 MAD Shift, mm	Rebound, *n* (%)
Isolated distal medial femur	17	−7.18 ± 6.53	11 (64.7)
Isolated proximal medial tibia	9	−1.56 ± 6.54	3 (33.3)
Combined femur and tibia	5	−1.80 ± 2.68	1 (20.0)
Overall	31	−4.68 ± 6.55	15 (48.4)

Negative MAD values indicate lateral deviation.

**Table 4:** Exploratory univariable and multivariable linear-regression analyses of continuous t1-to-t2 MAD rebound.

Predictor	Univariable *b*	95% CI	*p*	Multivariable *β*	95% CI	*p*
Sex (male vs. female)	0.329	−4.908 to 5.566	0.899	—	—	—
Isolated distal femoral plating (yes vs. no)	5.534	1.084 to 9.983	0.017 *	0.348	0.519 to 8.498	0.028 *
Age at t0, years	0.390	−1.568 to 2.349	0.687	—	—	—
Age at t1, years	−0.462	−2.116 to 1.192	0.572	—	—	—
Initial valgus magnitude, mm	−0.295	−0.616 to 0.027	0.071	—	—	—
Correction duration, months	−0.285	−0.554 to −0.016	0.039 *	—	—	**—**
MAD correction rate, mm/month	0.485	−0.817 to 1.787	0.452	—	—	—
Residual growth after removal, cm	0.621	0.232 to 1.010	0.003 *	0.458	0.180 to 0.918	0.005 *

Statistically significant values are in bold. * *p* ≤ 0.05.

## Data Availability

The original contributions presented in the study are included in the article material; further inquiries can be directed to the corresponding author.
